# The Effects of Dynamic Balance Training on Balance and Walking Function in Stroke Patients

**DOI:** 10.3390/healthcare14080985

**Published:** 2026-04-09

**Authors:** Jianhua Li, Jian Wang, Renxiu Bian

**Affiliations:** 1 College of Education, Zhejiang University, Hangzhou 310058, China; jianhua_li@zju.edu.cn (J.L.);; 2 Department of Rehabilitation Medicine, Sir Run Run Shaw Hospital, Zhejiang University School of Medicine, Hangzhou 310016, China

**Keywords:** stroke, dynamic balance, center of pressure (COP), walking function, electromyographic (EMG) activity

## Abstract

Background: Stroke-related impairments in balance and gait are among the most common and disabling sequelae, significantly limiting functional independence and increasing fall risk. This study investigated the effects of short-term dynamic balance training on balance and gait in post-stroke hemiplegic patients. Methods: In this randomized controlled pilot trial, 16 post-stroke hemiplegic patients (intervention group, *n* = 8; control group, *n* = 8; mean age ≈ 58 years; predominantly male) were assigned to either a control group receiving conventional rehabilitation or an intervention group receiving additional daily dynamic balance training using the Prokin-252 system (30 min/day, 5 days/week, 3 weeks). Primary outcome measures included balance performance (Berg Balance Scale, mini-BESTest, single-leg stance), center-of-pressure (COP) parameters, gait performance (Timed Up and Go Test), and surface electromyography (sEMG) activity. Results: Following the intervention, both groups demonstrated improvements; however, the intervention group showed significantly greater gains in balance and gait outcomes. Specifically, Berg Balance Scale scores improved significantly (*p* = 0.012), as did mini-BESTest scores (*p* = 0.004). Eyes-closed single-leg stance time increased significantly on both sides (*p* < 0.05). COP analysis revealed reductions in sway area and trajectory length under challenging conditions. sEMG analysis indicated increased activation of the affected-side gluteus medius. In terms of gait performance, the intervention group demonstrated greater improvements in Timed Up and Go Test performance (*p* = 0.002), dual-task walking, and gait phase symmetry. Conclusions: Supplementing conventional rehabilitation with dynamic balance training effectively enhances balance and gait function in post-stroke patients, potentially through improved neuromuscular control. The integration of sensor-based COP analysis and sEMG provides additional mechanistic insight into rehabilitation outcomes.

## 1. Introduction

Stroke remains one of the leading causes of mortality and long-term disability worldwide, ranking as the second leading cause of death and the third leading cause of disability-adjusted life years globally [1]. In China, the incidence and prevalence of stroke have continued to increase in recent decades, with epidemiological studies reporting a rise in prevalence from approximately 1.89% to over 2.58%, and disability rates reaching 70–80%, thereby imposing a substantial burden on healthcare systems and families [2]. Although advances in acute medical care have significantly improved survival rates, post-stroke functional impairment remains prevalent and often severe, particularly in the early rehabilitation phase.

Among post-stroke impairments, motor dysfunction—particularly deficits in balance and gait—is one of the most common and disabling sequelae [3]. Impaired balance and walking ability restrict activities of daily living, increase the risk of falls, and substantially reduce quality of life. Therefore, restoring balance and gait function is a central goal of post-stroke rehabilitation. The primary objective of stroke rehabilitation is to promote recovery of physiological and functional abilities through multidimensional interventions, ultimately enhancing activity participation and quality of life [4,5]. Balance function serves as a foundational component of motor recovery and largely determines the upper limit of functional independence and social reintegration after stroke [6]. Furthermore, previous studies have demonstrated that improvements in balance are strongly associated with enhanced gait performance and reduced fall risk in stroke populations [7].

Current exercise-based balance interventions for patients with stroke generally fall into two categories: (1) strengthening-oriented training aimed at enhancing limb support capacity, and (2) balance training using specialized equipment. The latter includes static weight-shifting training on stable surfaces and dynamic balance training on unstable surfaces. Although growing evidence suggests that dynamic balance training on unstable platforms can improve both balance and gait by engaging feedforward and feedback postural control mechanisms [8,9,10]. However, despite these demonstrated clinical benefits, most existing studies have primarily focused on functional outcomes such as clinical balance scales and walking speed, while providing limited insight into the underlying neuromuscular control mechanisms [11,12,13]. In addition, post-stroke hemiplegic patients often exhibit asymmetric muscle activation patterns between the affected and unaffected sides, which critically influence balance and gait recovery [14,15]. Although asymmetry has been widely reported, the task-specific modulation of neuromuscular activation and its relationship with postural control strategies remain insufficiently understood, particularly under dynamic balance conditions.

Importantly, few studies have simultaneously integrated sensor-based center-of-pressure (COP) analysis and surface electromyography (sEMG) to investigate both biomechanical and neuromuscular aspects of balance control. Such integrated approaches may provide a more comprehensive understanding of postural control mechanisms and offer objective evidence for optimizing rehabilitation strategies.

Therefore, this study aimed to investigate the effects of dynamic balance training on balance and gait function in patients with post-stroke hemiplegia, with a particular focus on sensor-based assessments of COP dynamics and sEMG-derived neuromuscular activation patterns. By integrating clinical functional outcomes with objective biomechanical and electrophysiological measurements, this study seeks to elucidate the neuromuscular mechanisms underlying dynamic balance training. Furthermore, this study is designed as an exploratory randomized controlled trial to provide preliminary evidence for more precise and individualized rehabilitation strategies in stroke patients.

## 2. Materials and Methods

### 2.1. Study Registration and Ethical Approval

This study was approved by the Ethics Committee of Sir Run Run Shaw Hospital, Zhejiang University School of Medicine (Approval No.: 2023-Research-0321). The trial was registered at the Chinese Clinical Trial Registry (ChiCTR) (Identifying number: ChiCTR2300070423). All participants provided written informed consent prior to participation.

### 2.2. Participants

This study employed a randomized single-blind controlled design. Patients hospitalized in the Department of Rehabilitation Medicine at Sir Run Run Shaw Hospital, Zhejiang University School of Medicine, between June 2024 and January 2025 were consecutively screened. Patients were included if they met the diagnostic criteria for stroke with hemiplegia and had a lower-limb Brunnstrom stage ≥ IV. Exclusion criteria included clinical deterioration, pre-existing residual motor dysfunction, or other neurological diseases. A total of 16 patients were enrolled.

#### 2.2.1. Inclusion Criteria

Participants were included if they met all of the following criteria: Diagnosis of stroke according to the diagnostic criteria revised at the Fourth National Academic Conference on Cerebrovascular Diseases in 1995, confirmed by cranial computed tomography (CT) or magnetic resonance imaging (MRI) [16]; Lower-limb Brunnstrom stage ≥ IV; Unilateral involvement, including first-ever stroke or a history of cerebral infarction without residual motor dysfunction; Clear consciousness and stable vital signs; Ability to understand and follow instructions from therapists or devices and to complete the required tasks; Standing balance level ≥ grade II according to the simplified three-level balance assessment; Ability to walk independently for at least 5 m.

#### 2.2.2. Withdrawal, Dropout, and Termination Criteria

(1) Withdrawal Criteria

Participants were withdrawn if: they were found not to meet the inclusion criteria after enrollment or adverse events occurred during the study that made continued participation inappropriate. Withdrawn cases were documented with reasons and excluded from efficacy analyses.

(2) Dropout Criteria

Participants were considered dropouts if they: voluntarily withdrew during the intervention; were discharged early, transferred to another department, or transferred to another hospital; or had incomplete data that precluded efficacy evaluation.

(3) Termination Criteria

**Figure 1:**
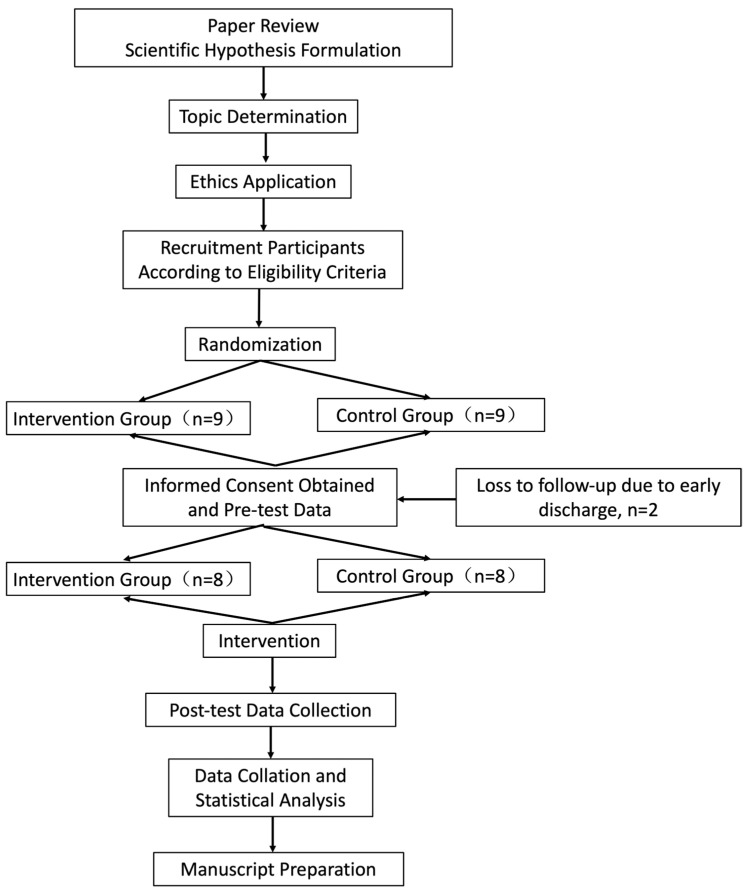
CONSORT flow diagram of participant recruitment, allocation, follow-up, and analysis.

The study was terminated for participants who experienced serious adverse events or adverse reactions that interfered with safety or efficacy evaluation. For dropouts and terminated participants, reasons were recorded at the time of discontinuation. Participants who completed more than two-thirds of the intervention sessions were included in efficacy analyses. All data were properly archived, and serious adverse events were reported to the ethics committee (Figure 1).

### 2.3. Randomization and Allocation Concealment

This study employed a randomized, single-blind controlled design and was conducted as an exploratory pilot trial. Participants were assigned identification numbers according to the order of enrollment. Randomization was performed using Microsoft Excel, and random numbers were generated using the function “=RANDBETWEEN(1, 16)” in WPS Spreadsheets (version 11.1.0.12763).

The randomization sequence was prepared by an independent third party who was not involved in participant recruitment or outcome assessment. The allocation sequence was concealed in sequentially numbered, opaque, sealed envelopes. The research coordinator opened the envelopes and informed participants of their group allocation.

To minimize potential bias, outcome assessments were conducted by fixed evaluators who were blinded to group assignment, and all statistical analyses were performed by an independent statistician.

No a priori sample size calculation or power analysis was performed due to the exploratory nature of this pilot study, and the sample size was determined based on case availability during the study period.

### 2.4. Intervention Protocols

**Figure 2:**
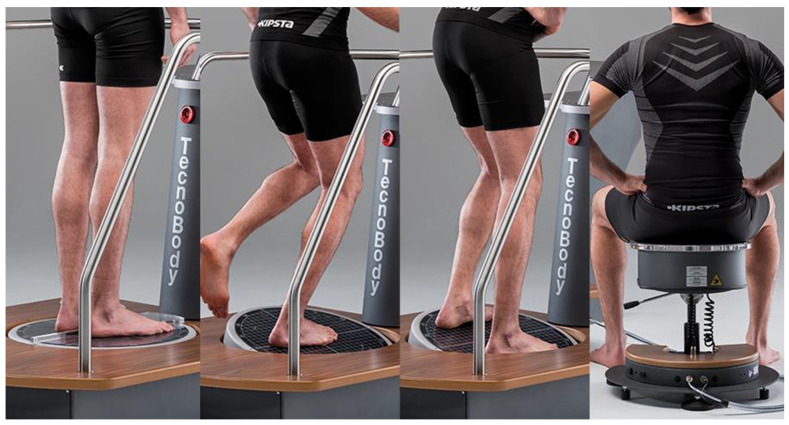
Prokin equipment.

Both groups received conventional rehabilitation therapy, which was individualized but kept as consistent as possible across groups. It included physical therapy (muscle strengthening, range-of-motion exercises, balance training, gait training), occupational therapy, hand function training, and physical agent modalities. The intervention group underwent dynamic balance training using the Tecnobody balance assessment and training system (model: PK252) (Figure 2). Training was administered once daily, five times per week, for 3 weeks, with each session lasting approximately 30 min. Training tasks included single-leg anterior–posterior and medial–lateral COP control, with difficulty progressively increased by adjusting resistance, frequency, and movement amplitude.

#### 2.4.1. Conventional Rehabilitation Training

Participants in both the intervention and control groups received conventional rehabilitation programs, including physical therapy, occupational therapy, hand function training, and physical agent modalities.

All interventions were individualized according to patient condition but were standardized in structure, frequency, and duration across groups to ensure comparability of treatment exposure.

The conventional rehabilitation program was delivered once daily, five days per week, for approximately 60 min per session. Each session followed a predefined structure consisting of: (1) 20–25 min of physical therapy (including muscle strengthening, range-of-motion exercises, balance training, and gait training), (2) 15–20 min of occupational therapy focusing on upper-limb and functional task training, and (3) 10–15 min of adjunct therapies, including hand function training and physical agent modalities (e.g., neuromuscular electrical stimulation or thermal therapy), depending on clinical indications.

To ensure consistency, all rehabilitation sessions were conducted by licensed therapists who followed a standardized treatment protocol developed by the rehabilitation department. Treatment intensity was maintained at a low-to-moderate level and adjusted according to patient tolerance, while preserving the overall structure and duration of the program.

In addition, a treatment log was maintained for each participant to document session duration, intervention components, and any minor adjustments, thereby facilitating adherence monitoring and ensuring consistency across participants.

#### 2.4.2. Dynamic Balance Training

Dynamic balance training was performed using the Tecnobody balance assessment and training system (model: PK252). During training, the system’s mechanical lock was removed. One foot was placed on the balance platform, while the contralateral foot remained on the ground. Participants were instructed to maintain an upright trunk posture and control the balance platform primarily through movements of the lower limb or ankle to guide the on-screen cursor according to task instructions.

Training tasks (Table 1) included single-leg anterior–posterior COP control (single-axis proprioceptive assessment/balance/skill), medial–lateral COP control (single-axis proprioceptive assessment/balance/skill), diagonal movements (light task), and circular COP control within a defined range (multi-axis proprioceptive assessment). Training difficulty and intensity were progressively increased by adjusting resistance, frequency, movement amplitude, extending training duration, and shortening inter-task intervals.

Each task lasted 1–2 min, with a total session duration of approximately 30 min (±5 min), including one rest period. The intervention was administered once daily, five times per week, for 3 weeks. Exercise intensity for patients with stroke was maintained at a low-to-moderate level.

### 2.5. Outcome Measures

Outcome measures were categorized into three domains: (1) balance function, (2) gait and walking performance, and (3) neuromuscular activity assessed by surface electromyography (sEMG).

(1) Balance function

Balance performance was assessed using single-leg stance tests. Both affected and unaffected sides were tested under eyes-open and eyes-closed conditions. Participants stood upright with hands on hips, standing on the test leg while the contralateral leg was lifted off the ground. Timing began when the lifted foot left the ground and ended when the foot touched down or when substantial postural sway occurred.

Clinical balance scales included the Berg Balance Scale (BBS) and the Mini Balance Evaluation Systems Test (mini-BESTest). The BBS consists of 14 items (e.g., standing, sitting, functional reach, single-leg stance), each scored from 0 to 4, yielding a maximum score of 56. Higher scores indicate better balance performance. The BBS has been validated as a reliable and practical tool for assessing balance in patients with stroke [17].

The mini-BESTest evaluates balance across four domains: anticipatory postural adjustments, reactive postural control, sensory orientation, and dynamic gait. It includes 14 items scored from 0 to 2, with a maximum score of 28. The Chinese version of the mini-BESTest has demonstrated good reliability and validity [8].

In addition, center-of-pressure (COP) metrics were collected during 30 s bipedal standing tasks using a balance force platform (K-Force, KINVENT, France). Four standing conditions were tested: eyes-open with feet apart, eyes-open with feet together, eyes-closed with feet apart, and eyes-closed with feet together. Outcome variables included COP area, longitudinal and lateral axes of the COP confidence ellipse, weight distribution, COP total excursion, mean COP position, and footprint parameters.

(2) Gait and Walking Function

Walking ability was assessed using the Timed Up and Go Test (TUGT). Participants were instructed to stand up from a standardized chair, walk 3 m, turn around, return, and sit down. Two trials were performed, and the mean time was recorded. Participants were given standardized instructions and demonstrations before testing, and safety supervision was provided without assistance during the test.

Three-dimensional gait analysis was conducted during a 5 m straight or shuttle walking task using the Ariel Performance Analysis System (APAS). Two cameras simultaneously recorded motion from the frontal and left lateral views. A calibration frame was recorded before each session. Videos were imported into APAS software (version 14.3) for frame-by-frame digitization, smoothing, and calculation of kinematic variables, including center of mass, velocity, acceleration, displacement, joint angles, angular velocity, and power.

Eleven anatomical landmarks were digitized according to previous studies, including the ankle, knee, hip, shoulder, midpoint of the hips, midpoint of the shoulders, and head.

(3) Surface Electromyography

Surface electromyography (sEMG) signals were recorded during the 30 s standing tasks and gait tasks using a 16-channel wireless sEMG system (Trigno, Delsys, Natick, MA, USA). Thirteen electrodes were placed over the muscle bellies of major trunk and lower-limb muscles on the affected side and bilaterally where appropriate, and secured with elastic bandages.

Recorded muscles included the multifidus, erector spinae, and gluteus medius on the affected side; and the soleus, medial gastrocnemius, tibialis anterior, rectus femoris, and biceps femoris on both affected and unaffected sides. Mean EMG amplitude and integrated EMG (iEMG) were calculated. Mean EMG reflected average signal amplitude, while iEMG represented the total rectified and smoothed EMG area per unit time, indicating the overall motor unit recruitment during muscle activity.

### 2.6. Statistical Analysis

#### 2.6.1. Data Processing

Gait videos were processed using APAS software for segmentation, digitization, three-dimensional modeling, and data computation. For gait analysis, one complete gait cycle was extracted from initial contact to subsequent contact of the same foot. Gait phases were further subdivided according to the Rancho Los Amigos gait cycle classification, with the affected limb defined as the reference limb.

sEMG data were processed in MATLAB (The MathWorks, Inc., Natick, MA, USA, R2024a). Signals were high-pass filtered at 35 Hz, demeaned, full-wave rectified, down-sampled, low-pass filtered at 40 Hz, and normalized. Non-negative matrix factorization was applied to reduce sEMG dimensionality into motor modules. Neuromuscular complexity was quantified by determining the number of modules required to reconstruct the original EMG signals above a predefined variance threshold. Mean EMG and iEMG values were also computed.

#### 2.6.2. Statistical Methods

Statistical analyses were performed using SPSS version 25.0 (IBM Corp., Armonk, NY, USA). Continuous variables are presented as mean ± standard deviation, and categorical variables as percentages. Repeated-measures analysis of variance (RM-ANOVA) was used to assess differences between groups over time. In addition to *p*-values, effect sizes were calculated for key outcome variables to evaluate the magnitude of intervention effects. Baseline between-group comparisons were conducted using independent-samples *t*-tests for normally distributed data or Mann–Whitney U tests for non-normally distributed data. Within-group comparisons were performed using paired-samples *t*-tests or Wilcoxon signed-rank tests as appropriate.

Normality was assessed using the Shapiro–Wilk test. All statistical tests were two-tailed, and a *p*-value < 0.05 was considered statistically significant.

No formal correction for multiple comparisons was applied due to the exploratory nature of this pilot study; therefore, results should be interpreted with caution.

## 3. Results

### 3.1. Baseline Characteristics

As shown in Table 2, no significant differences were observed between the intervention and control groups at baseline across demographic and clinical variables (*p* > 0.05), indicating good comparability between groups.

The median time since stroke onset was 10 days in the intervention group and 20 days in the control group.

### 3.2. Changes in Balance Function

Significant group × time interaction effects were observed for the Berg Balance Scale (BBS) (F = 8.438, *p* = 0.012), indicating a greater improvement in the intervention group. A significant main effect of time was also observed for both BBS (F = 9.223, *p* = 0.009) and mini-BESTest (F = 11.667, *p* = 0.004).

Post hoc analysis revealed significant within-group improvements in the intervention group for BBS (*p* = 0.013) and mini-BESTest (*p* = 0.002), indicating large effect sizes. In contrast, no significant within-group improvements were observed in the control group.

For single-leg stance performance, significant improvements over time were observed across conditions (*p* < 0.05), with the intervention group showing greater improvements, particularly under eyes-closed conditions (Table 3).

**Figure 3:**
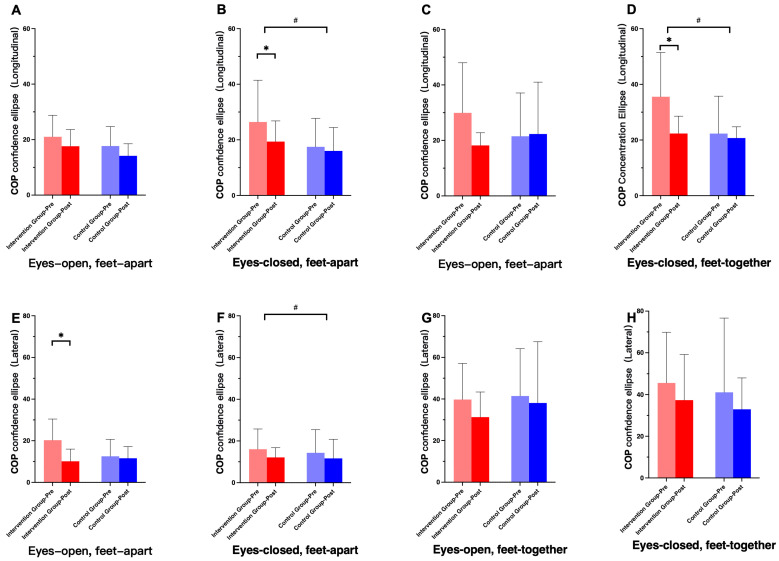
Changes in the longitudinal and lateral axes of the COP confidence ellipse before and after intervention. (**A**–**D**) represent the lengths of the longitudinal axis, and (**E**–**H**) represent the lengths of the transverse axis. (**A**,**E**): eyes open, feet apart; (**B**,**F**): eyes closed, feet apart; (**C**,**G**): eyes open, feet together; (**D**,**H**): eyes closed, feet together. Note: * indicates a significant interaction effect; # indicates a significant main effect of time.

COP analysis demonstrated significant reductions in sway parameters following intervention, particularly under eyes-closed conditions. Significant main effects of time were observed for both the longitudinal and lateral axes of the COP confidence ellipse (*p* < 0.05) (Figure 3).

**Figure 4:**
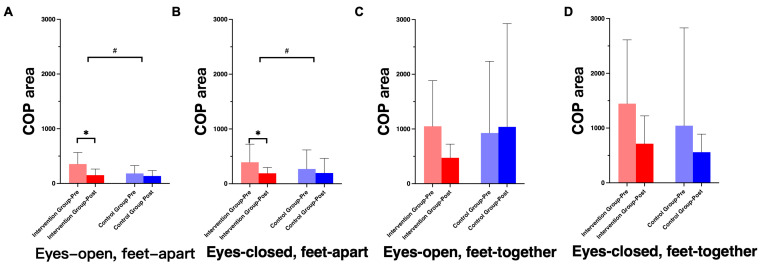
Changes in center-of-pressure (COP) area during standing tasks before and after intervention. (**A**): eyes open, feet apart; (**B**): eyes closed, feet apart; (**C**): eyes open, feet together; (**D**): eyes closed, feet together. Note: * indicates a significant interaction effect; # indicates a significant main effect of time.

Post hoc analysis indicated that the intervention group showed significant reductions in COP trajectory and area under more challenging balance conditions, whereas changes in the control group did not reach statistical significance (Figure 4).

**Figure 5:**
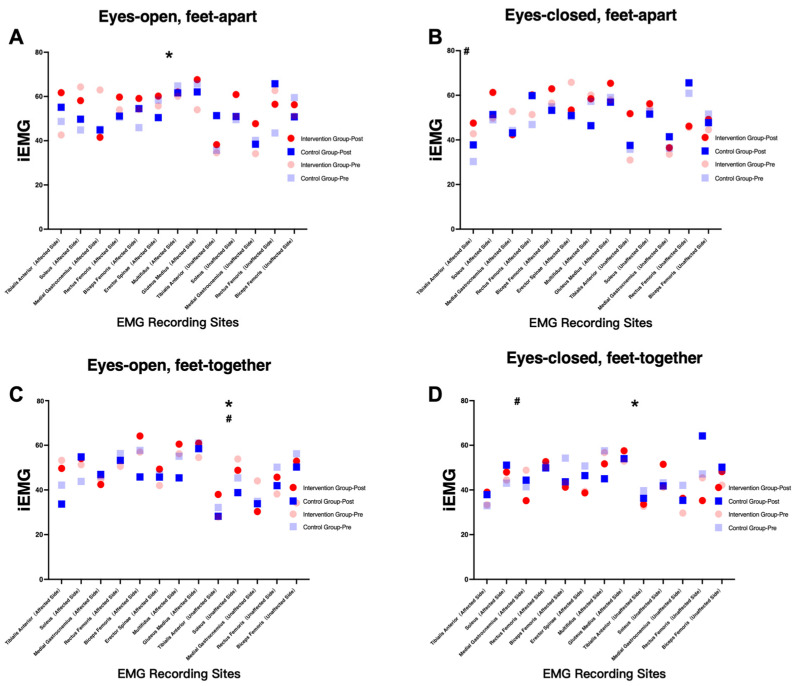
Changes in integrated EMG (iEMG) of selected muscles during standing balance tasks before and after intervention. (**A**): eyes open, feet apart; (**B**): eyes closed, feet apart; (**C**): eyes open, feet together; (**D**): eyes closed, feet together. Note: * indicates a significant interaction effect; # indicates a significant main effect of time.

**Figure 6:**
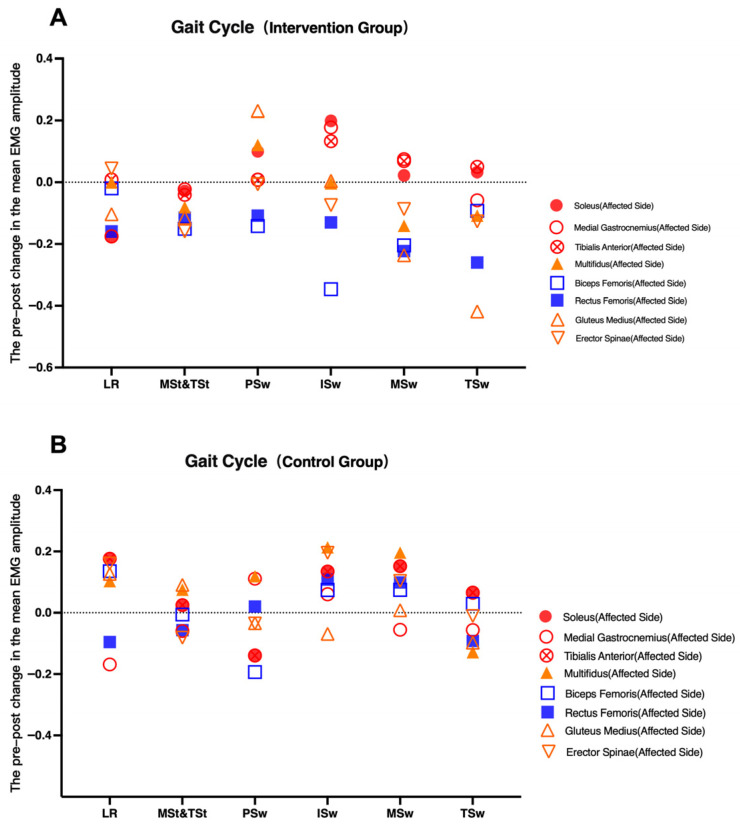
Changes in affected-side muscle iEMG across gait phases before and after intervention. (**A**): Gait Cycle (Intervention Group); (**B**): Gait Cycle (Control Group).

**Figure 7:**
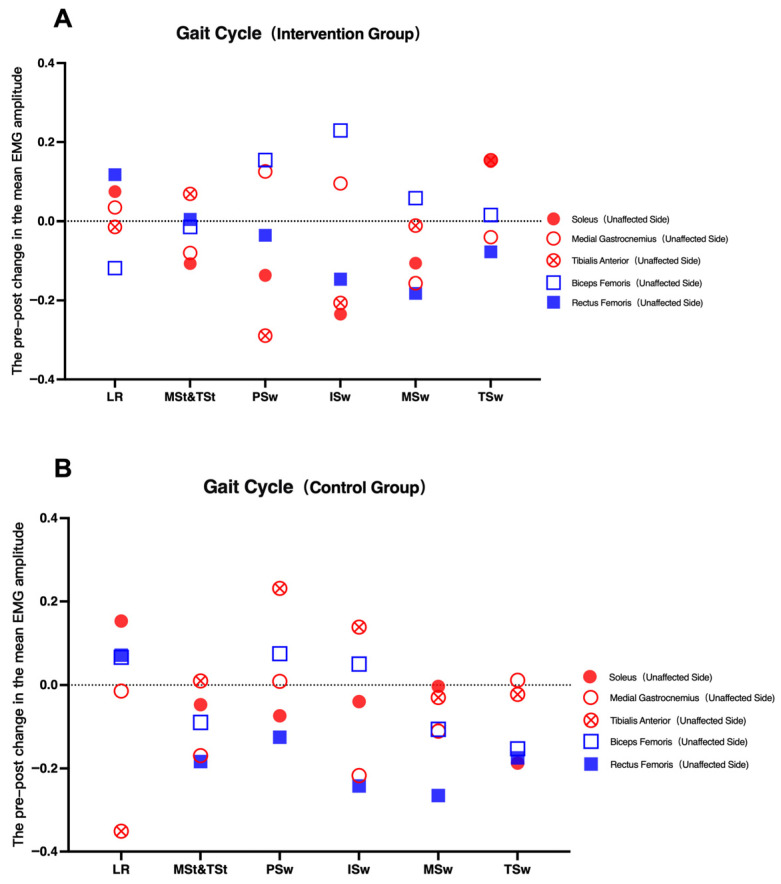
Changes in unaffected-side muscle iEMG across gait phases before and after intervention. (**A**): Gait Cycle (Intervention Group); (**B**): Gait Cycle (Control Group).

**Figure 8:**
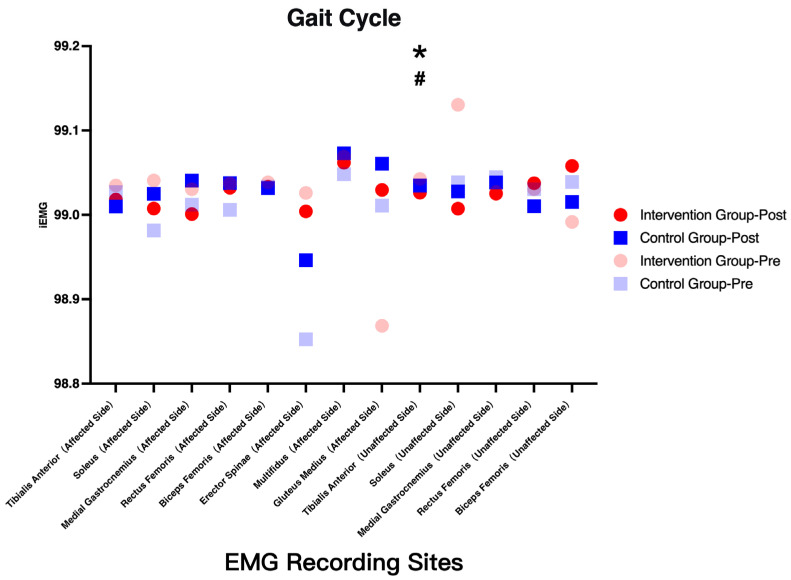
Changes in integrated EMG (iEMG) of selected muscles across gait phases before and after intervention. Note: * indicates a significant interaction effect; # indicates a significant main effect of time.

sEMG analysis revealed increased activation of the affected-side gluteus medius in the intervention group (*p* < 0.05), suggesting enhanced neuromuscular engagement. However, this increase should be interpreted cautiously, as it may reflect either improved motor control or compensatory activation strategies (Figure 5, Figure 6, Figure 7 and Figure 8).

### 3.3. Changes in Gait Function

Significant improvements in gait performance were observed following the intervention. Specifically, TUGT time decreased significantly in the intervention group (*p* = 0.002), indicating improved functional mobility (Table 4A,B).

Spatiotemporal analysis revealed significant increases in gait speed (*p* = 0.021) and step length (*p* = 0.032). Additionally, gait phase analysis showed an increase in the affected-side single-support phase and a reduction in the double-support phase (*p* < 0.05), suggesting improved gait symmetry and stability (Table 4C–E).

## 4. Discussion

In this randomized controlled trial, patients with post-stroke hemiplegia underwent a short-term dynamic balance training program during hospitalization. Improvements in balance and gait function were observed in both groups, with more pronounced gains in the intervention group. Specifically, the main findings of this study can be summarized as follows: (1) balance scale scores, including the Berg Balance Scale and the mini-BESTest, improved in both groups, with significant within-group increases observed in the intervention group; (2) single-leg stance performance under both eyes-open and eyes-closed conditions improved after intervention, with significant improvements under eyes-closed conditions in the intervention group and increasing trends under more challenging conditions; (3) improvements in center-of-pressure (COP) parameters were observed, particularly under eyes-closed conditions, indicating enhanced postural stability; (4) increased activation of the affected-side gluteus medius was observed during balance tasks; and (5) gait-related outcomes, including gait stability and temporal phase distribution, improved following intervention.

Collectively, these findings suggest that dynamic balance training provides additional benefits beyond conventional rehabilitation, particularly in enhancing both functional performance and neuromuscular coordination.

Post-stroke hemiplegic patients commonly exhibit abnormal muscle tone, impaired voluntary movement, and sensory deficits, particularly proprioceptive impairments, which reduce weight-bearing capacity and postural stability [10,11]. These deficits contribute to balance dysfunction, abnormal posture, impaired gait, and an increased risk of falls. Dynamic balance training using unstable platforms aims to enhance anticipatory activation of postural muscles prior to target movements and to improve compensatory postural adjustments in response to perturbations [12,13,14].

These findings are consistent with previous studies demonstrating that unstable surface training can improve postural control and gait performance by facilitating sensorimotor integration [18]. However, unlike prior studies that primarily focused on clinical outcomes, the present study further demonstrates that these functional improvements are accompanied by measurable changes in COP dynamics and neuromuscular activation patterns.

Specifically, by visualizing the center of pressure and training patients to actively control COP displacement, dynamic balance training may enhance both visual and proprioceptive feedback, thereby promoting more efficient control of the body’s center of mass. This interpretation is consistent with established models of postural control, which emphasize the integration of sensory inputs and motor outputs for maintaining equilibrium [18].

During COP stabilization, postural muscle activation follows specific coordination patterns. Krishnamoorthy et al. proposed that the central nervous system utilizes a limited set of control variables to coordinate multiple muscles and generate appropriate COP displacement [19]. In the present study, surface electromyography (sEMG) was used to assess neuromuscular activation patterns, with a particular focus on integrated EMG (iEMG), which reflects both the magnitude and temporal summation of muscle activity [20].

Notably, increased activation of the affected-side gluteus medius was observed in the intervention group across multiple balance conditions. This finding may indicate improved recruitment of hip stabilizing muscles, which play a critical role in maintaining lateral stability during both static and dynamic tasks. Previous studies have shown that motor module reorganization and improved intermuscular coordination are associated with better functional recovery after stroke [21].

However, it is important to interpret these findings with caution. Increased iEMG activity does not necessarily indicate improved neuromuscular efficiency; rather, it may also reflect compensatory activation strategies or increased co-contraction aimed at stabilizing posture. This is consistent with previous research suggesting that post-stroke motor recovery often involves both restitution and compensation mechanisms [22].

Across different standing conditions, distinct patterns of muscle activation were observed. Under less challenging conditions (eyes open, feet apart), the intervention group demonstrated more balanced activation between anterior and posterior muscle groups, whereas the control group exhibited a tendency toward compensatory activation of the unaffected side. Under more challenging conditions (eyes closed, reduced base of support), both groups showed reduced overall EMG amplitude; however, the intervention group maintained relatively greater activation of trunk and hip stabilizers.

These findings suggest that dynamic balance training may facilitate a redistribution of neuromuscular activity toward more functionally relevant muscle groups, particularly those involved in proximal stabilization. This observation aligns with previous studies highlighting the importance of hip strategy and proximal control in maintaining balance under unstable conditions [23,24].

From a clinical perspective, the magnitude of improvement observed in balance outcomes appears to exceed previously reported minimal clinically important differences (MCID) for the Berg Balance Scale [25]. Similarly, improvements in gait-related parameters may correspond to clinically meaningful changes in walking ability [26]. These findings suggest that the observed improvements are not only statistically significant but also functionally meaningful.

Such improvements may translate into reduced fall risk and enhanced independence in activities of daily living, which are key goals of stroke rehabilitation. These results are consistent with systematic reviews indicating that targeted rehabilitation interventions can significantly improve functional mobility and participation in stroke populations [27].

Several limitations of this study should be acknowledged. First, the sample size was relatively small (*n* = 16), and no a priori sample size calculation was performed, which limits the statistical power and generalizability of the findings. Therefore, the results should be interpreted as exploratory.

Second, this was a single-center study, which may limit external validity. Third, no long-term follow-up was conducted, and thus the sustainability of the observed improvements remains unclear.

In addition, a large number of outcome measures and statistical comparisons were performed without formal correction for multiple comparisons, which may increase the risk of type I error. Finally, although sEMG provides valuable information regarding muscle activation, it does not directly reflect muscle force or coordination efficiency, and the interpretation of EMG findings should therefore be approached with caution [20].

Future studies should include larger sample sizes, multi-center designs, and longer follow-up periods to validate and extend these findings. Additionally, further research is needed to explore the dose–response relationship of dynamic balance training and to integrate more advanced biomechanical and neurophysiological assessments to better understand the mechanisms underlying motor recovery after stroke [24,27].

## 5. Conclusions

This randomized controlled trial demonstrated that short-term dynamic balance training during hospitalization can effectively improve balance function and standing postural stability in patients with post-stroke hemiplegia, as evidenced by both clinical assessments and center-of-pressure (COP)-based measures. Moreover, the observed increase in activation of the affected-side gluteus medius indicates that dynamic balance training may influence neuromuscular engagement during postural control tasks; however, this finding should be interpreted cautiously, as it may reflect both adaptive and compensatory motor strategies.

By incorporating synchronized surface electromyography (sEMG) recordings of trunk and lower-limb muscles during balance and gait tasks, the present study provides additional insight into the potential neuromuscular adaptations associated with dynamic balance training. This integrative assessment approach extends beyond conventional clinical evaluation and contributes to a more comprehensive understanding of post-stroke motor recovery.

Overall, the findings support the use of dynamic balance training as an effective adjunct to conventional rehabilitation for improving balance and gait performance in post-stroke populations. Notably, the improvements observed in this study may have practical clinical relevance, suggesting potential benefits for functional independence and fall risk reduction.

Nevertheless, the conclusions of this study should be interpreted within the context of its limitations, including the small sample size, single-center design, and lack of long-term follow-up. In addition, the exploratory nature of the study highlights the need for further research to clarify the underlying mechanisms and to determine optimal training parameters.

Future studies incorporating larger, multi-center samples, longitudinal follow-up, and more rigorous experimental designs are warranted to confirm these findings and to further refine individualized rehabilitation strategies based on biomechanical and neurophysiological assessments.

## Figures and Tables

**Table 1:** Prokin Intervention plan.

Tasks	Affected and Unaffected Sides	Direction	Time	Repetitions
single-axis proprioceptive assessment	Both	anterior–posterior	1 min	2
		medial–lateral	1 min	2
balance/skill	Both	anterior–posterior	2 min	2
		medial–lateral	2 min	2
multi-axis proprioceptive assessment	Both		2 min	2
light task	Both		2 min	1

**Table 2:** Baseline characteristics of participants.

Characteristic	Intervention Group (*n* = 8)	Control Group (*n* = 8)	*p* Value
Sex			
Male, *n* (%)	7 (87.5)	7 (87.5)	
Female, *n* (%)	1 (12.5)	1 (12.5)	
Age (years)	57.17 ± 11.96	58.40 ± 10.41	0.861
Height (cm)	166.66 ± 6.97	168.46 ± 11.58	0.712
Weight (kg)	70.12 ± 6.44	70.76 ± 12.45	0.899
Waist Circumference (kg)	91.90 (84.8, 92.3)	90.65 (88.8, 95.5)	0.600
Hip Circumference (kg)	94.58 ± 4.46	96.63 ± 5.85	0.444
Body Mass Index (kg/m^2^)	25.29 ± 2.03	24.59 ± 2.46	0.298
Duration (days)	10.00 (9.0, 71.0)	20.00 (9.5, 309.0)	0.512
Non-First Stroke, *n* (%)	1 (12.5)	1 (12.5)	
Hemiplegic Side			
Right, *n* (%)	5 (62.5)	5 (62.5)	
Left, *n* (%)	3 (37.5)	3 (37.5)	
Lower Limb Brunnstrom Stage	4.88 ± 0.35	4.63 ± 0.74	0.411
Number of Rehabilitation Items	9.00 (6.0, 9.0)	8.00 (8.0, 9.0)	0.864
Days in Trial	15.13 ± 6.73	13.00 ± 1.51	0.410
Intervention Sessions	12.13 ± 5.46		
Physical Activity Level (MET/day)	1.01 (1.0, 1.1)	1.02 (1.0, 1.0)	1
Sedentary Behavior (%)	0.87 ± 0.10	0.90 ± 0.06	0.593
Light PA (%)	0.12 ± 0.09	0.10 ± 0.06	0.617
Moderate PA (%)	0.00 (0.0, 0.0)	0.00 (0.0, 0.0)	0.745

Note: Significance levels are denoted as follows: *p* < 0.05 indicates a statistically significant difference; *p* < 0.01 indicates a highly significant statistical difference; *p* < 0.001 indicates an extremely significant statistical difference. Data are presented as Mean ± SD, Median (IQR), or *n* (%) as appropriate. PA = Physical Activity. MET = Metabolic Equivalent of Task.

**Table 3:** Balance and functional performance outcomes.

Measure	Group	Pre	Post	Interaction F	Interaction P	Time F	Time P	Group F	Group P
Berg Balance Scale	Intervention	47.88 ± 4.97	53.50 ± 1.60	8.438	0.012 *	9.223	0.009 **	0.226	0.642
	Control	49.25 ± 7.72	49.38 ± 7.82						
Mini BESTest	Intervention	21.63 ± 2.50	24.13 ± 2.36	1.296	0.274	11.667	0.004 **	2.497	0.136
	Control	19.38 ± 4.21	20.63 ± 5.32						
Functional Reach	Intervention	29.26 ± 11.38	36.48 ± 8.36	3.606	0.080	1.966	0.184	1.023	0.330
	Control	27.41 ± 8.78	27.89 ± 9.44						
Sitting Reach (Unaffected)	Intervention	23.50 ± 3.95	33.35 ± 5.68	8.280	0.021 *	40.077	0.000 ***	8.757	0.018 *
	Control	23.29 ± 7.02	26.44 ± 3.53						
Sitting Reach (Affected)	Intervention	24.40 ± 4.01	30.97 ± 5.91	1.136	0.318	5.261	0.051	5.524	0.047 *
	Control	19.55 ± 7.06	21.95 ± 5.05						
Single-Leg Stance EO (Affected)	Intervention	3.16 ± 3.65	10.06 ± 10.71	1.102	0.316	5.225	0.043 *	0.119	0.737
	Control	4.11 ± 3.84	8.33 ± 6.73						
Single-Leg Stance EO (Unaffected)	Intervention	7.56 ± 7.78	18.49 ± 19.65	1.061	0.325	5.471	0.039 *	0.176	0.683
	Control	14.72 ± 17.93	17.05 ± 18.50						
Single-Leg Stance EC (Affected)	Intervention	1.04 ± 0.59	1.72 ± 0.64	1.151	0.303	6.805	0.022 *	0.639	0.438
	Control	1.42 ± 0.51	1.70 ± 0.49						
Single-Leg Stance EC (Unaffected)	Intervention	1.88 ± 1.04	3.64 ± 2.41	5.060	0.042 *	9.304	0.009 **	1.920	0.189
	Control	4.96 ± 4.19	5.23 ± 4.69						

Note: Data are presented as Mean ± Standard Deviation. EO = eyes open; EC = eyes closed. Significance levels: *p* < 0.05 *, *p* < 0.01 **, *p* < 0.001 ***.

**Table 4:** (**A**) Gait stability outcomes (Mini-BESTest). (**B**) Functional mobility outcomes (TUG and dual-task TUG). (**C**) Spatiotemporal gait parameters. (**D**) Gait phase distribution (affected side). (**E**) Gait phase distribution (Unaffected Side).

(A)									
Outcome		Group		Pre	Post	Interaction (P)		Time (P)	Group (P)
Walking with Speed Changes		Intervention		1.75 ± 0.46	2.00 ± 0.00	0.149		0.149	0.506
		Control		1.75 ± 0.46	1.75 ± 0.46				
Walking with Head Turns		Intervention		1.50 ± 0.53	1.75 ± 0.46	0.010 *		0.081	0.349
		Control		1.38 ± 0.52	1.50 ± 0.46				
(**B**)									
**Outcome**	**Group**	**Pre**	**Post**	**Interaction F**	**Interaction P**	**Time F**	**Time P**	**Group F**	**Group P**
TUGT Time (s)	Intervention	15.94 ± 4.38	12.90 ± 3.89	0.921	0.355	15.253	0.002 *	0.105	0.751
	Control	14.80 ± 2.55	12.97 ± 2.08						
Dual-Task TUG (s)	Intervention	14.21 ± 4.30	11.81 ± 3.55	0.836	0.377	16.354	0.001 **	0.205	0.658
	Control	14.57 ± 3.15	13.05 ± 2.79						
(**C**)									
**Outcome**	**Group**	**Pre**	**Post**	**Interaction F**	**Interaction P**	**Time F**	**Time P**	**Group F**	**Group P**
Gait Cycle Time	Intervention	1.47 ± 0.27	1.33 ± 0.10	0.231	0.638	3.350	0.089	3.630	0.077
	Control	1.29 ± 0.21	1.21 ± 0.16						
Cadence	Intervention	41.95 ± 6.60	45.37 ± 3.50	0.058	0.814	2.715	0.122	3.674	0.076
	Control	47.86 ± 9.25	50.41 ± 6.47						
Walking Speed	Intervention	2.10 ± 0.71	2.51 ± 0.64	0.133	0.721	6.732	0.021 *	0.226	0.642
	Control	2.18 ± 0.82	2.73 ± 0.82						
Stride Length	Intervention	82.75 ± 22.78	91.20 ± 17.22	0.355	0.561	5.697	0.032 *	0.016	0.900
	Control	78.47 ± 27.64	92.54 ± 29.80						
Step Width	Intervention	15.47 ± 4.32	14.54 ± 5.24	0.393	0.541	0.078	0.784	0.334	0.572
	Control	15.88 ± 3.80	16.24 ± 3.08						
(**D**)									
**Outcome**	**Group**	**Pre**	**Post**	**Interaction F**	**Interaction P**	**Time F**	**Time P**	**Group F**	**Group P**
Stance Phase	Intervention	71 ± 7	68 ± 5	0.496	0.493	4.158	0.061	0.078	0.784
	Control	71 ± 5	70 ± 6						
Single Support	Intervention	28 ± 4	32 ± 5	2.385	0.145	8.236	0.012 *	1.060	0.321
	Control	31 ± 3	32 ± 4						
Double Support	Intervention	43 ± 7	37 ± 4	1.833	0.197	10.533	0.006 *	0.189	0.671
	Control	40 ± 5	38 ± 4						
Swing Phase	Intervention	29 ± 7	32 ± 5	0.496	0.493	4.158	0.061	0.078	0.784
	Control	29 ± 5	30 ± 6						
(**E**)									
**Outcome**	**Group**	**Pre**	**Post**	**Interaction F**	**Interaction P**	**Time F**	**Time P**	**Group F**	**Group P**
Stance Phase	Intervention	72 ± 4	68 ± 6	2.156	0.164	6.892	0.020 *	0.707	0.414
	Control	69 ± 3	68 ± 4						
Single Support	Intervention	29 ± 7	31 ± 6	0.018	0.896	1.414	0.254	0.005 *	0.942
	Control	29 ± 5	30 ± 6						
Double Support	Intervention	43 ± 7	38 ± 5	1.080	0.316	8.478	0.011 *	0.363	0.556
	Control	40 ± 5	38 ± 4						
Swing Phase	Intervention	28 ± 4	31 ± 6	1.042	0.325	5.272	0.038	1.402	0.256
	Control	31 ± 3	32 ± 4						

Note: Data are presented as mean ± standard deviation. Significance levels: *p* < 0.05 *, *p* < 0.01 **. ANOVA = analysis of variance. The “Time × Group” column represents the interaction effect. Mini-BESTest = Mini Balance Evaluation Systems Test. TUG = Timed Up and Go. Dual-Task TUG indicates performance under cognitive or motor interference.

## Data Availability

The datasets generated and analyzed during the current study are not publicly available due to privacy and ongoing analysis, but are available from the corresponding author on reasonable request.
